# Indocyanine green near-infrared fluorescence angiography in free parascapular flaps: Distal flap perfusion assessment in a retrospective study of 112 cases

**DOI:** 10.1016/j.jpra.2026.05.026

**Published:** 2026-05-20

**Authors:** Leonard Knoedler, Benjamin Thomas, Simon Mayer, Benedikt Geldner, Samuel Knoedler, Syena Moltaji, Alexandre G. Lellouch, Felix Struebing, Gabriel Hundeshagen, Ulrich Kneser, Florian Falkner

**Affiliations:** a Division of Plastic Surgery, Department of Surgery, Yale New Haven Hospital, Yale School of Medicine, New Haven, CT, USA; b Department of Oral and Maxillofacial Surgery, Berlin Institute of Health, Charité—Universitätsmedizin Berlin, Humboldt-Universität zu Berlin, Berlin, Germany; c Department of Hand, Plastic and Reconstructive Surgery, Burn Center, BG Trauma Center Ludwigshafen, University of Heidelberg, Ludwig-Guttmann-Strasse 13, 67071 Ludwigshafen, Germany; d Department of Hand and Plastic and Surgery, Ruprecht-Karls-University Heidelberg, Heidelberg, Germany; e Department of Plastic Surgery and Hand Surgery, Klinikum Rechts der Isar, Technical University of Munich, Munich, Germany; f Division of Plastic Surgery, University Health Network, University of Toronto, Toronto, Ontario, Canada; g Service de Chirurgie Plastique, Hôpital Européen Georges Pompidou, Assistance Publique-Hôpitaux de Paris (APHP), Université Paris Descartes, Paris, France; h Division of Plastic and Reconstructive Surgery, Massachusetts General Hospital, Harvard Medical School, Boston, MA, USA; i Vascularized Composite Allotransplantation Laboratory, Center for Transplantation Sciences, Massachusetts General Hospital, Harvard Medical School, Boston, MA, USA

**Keywords:** Free parascapular flap, PSC, Indocyanine-green fluorescence angiography, ICG, Near-infrared indocyanine green video angiography, ICG-NIR-VA, Flap surgery, Reconstructive surgery

## Abstract

**Background:**

Reliable perfusion of the distal portion of free parascapular (PSC) flaps is essential for successful lower extremity reconstruction. We hypothesized that intraoperative indocyanine green near-infrared video angiography (ICG-NIR-VA) enables reliable assessment of distal flap perfusion, thereby supporting microsurgical decision-making and reducing the risk of distal flap necrosis.

**Methods:**

We conducted a retrospective analysis of all free parascapular (PSC) flaps performed for lower extremity reconstruction between January 2015 and December 2024. Patients were non-randomly assigned to intraoperative perfusion assessment using indocyanine green near-infrared video angiography (ICG-NIR-VA; study group, n = 45) or conventional clinical evaluation (control group, n = 67). In both groups, inadequately perfused flap segments were trimmed intraoperatively at the surgeon’s discretion. Primary endpoints were distal or total flap necrosis, while secondary outcomes included reoperation rates and postoperative length of hospital stay (POLHS.

**Results:**

Intraoperative use of ICG-NIR-VA guided PSC flap design by identifying poorly perfused distal flap portions in 15 of 45 cases (33%), all of which resulted in uncomplicated postoperative courses. Partial flap necrosis occurred in 3 of 45 flaps (6.7%) in the ICG-NIR-VA group compared to 12 of 67 flaps (18.0%) in the control group (p = 0.09). Overall, intraoperative ICG-NIR-VA showed a strong correlation with postoperative outcomes, reflected by a negative predictive value of 98%. Furthermore, ICG-NIR-VA–assisted reconstructions required fewer recipient-site revision surgeries (9/45 [20.0%] vs. 24/67 [35.8%], p = 0.10) and showed a non-significant trend toward a shorter postoperative length of hospital stay (19 ± 10 vs. 21 ± 11 days, p = 0.40).

**Conclusions:**

Our findings suggest that ICG-NIR-VA may support intraoperative decision-making and may be associated with improved clinical outcomes, although several observed differences did not reach statistical significance.

## Introduction

As described by Nassif et al. in 1982, the free parascapular (PSC) flap has become an established reconstructive option, with reported success rates exceeding 90%.^1^, ^2^, ^3^, ^4^ Its reliable vascular anatomy and versatility have contributed to its broad applicability across reconstructive indications.^5^, ^6^, ^7^ However, the axial vascular pattern of the PSC flap may predispose distal flap segments to hypoperfusion, highlighting the importance of precise intraoperative flap planning. In our experience, there can exist an inexplicable inconsistency in vascular blood supply in flap lengths larger than 30 cm. Over the past two decades, indocyanine green near-infrared video angiography (ICG-NIR-VA) has emerged as a valuable tool for intraoperative assessment of tissue perfusion and flap viability.^8^, ^9^, ^10^, ^11^, ^12^, ^13^ This technique enables real-time visualization of microvascular blood flow and supports intraoperative decision-making.^14^ A recent review of 73 studies demonstrated that ICG-based imaging facilitates perforator identification in thin flaps (<20 mm) and assists in the evaluation of intraoperative microcirculation and perfusion.^15^ Despite these promising findings, studies specifically examining the role of ICG-NIR-VA in free PSC flap surgery remain limited.^1^^,^^16^ Consequently, evidence guiding the standardized implementation of this technology in PSC reconstruction is lacking. The aim of this retrospective study was to evaluate clinical outcomes and complication rates following free PSC flap reconstruction with adjunctive ICG-NIR-VA. By providing objective perfusion assessment, this approach may enhance intraoperative decision-making and optimize flap design, ultimately improving outcomes in lower extremity defect reconstruction.

## Methods

### Patient population

We retrospectively followed-up all patients who underwent free PSC flap lower extremity reconstruction at our institution between January 2015 and December 2024. Due to the lack of randomization in our study, the attending surgeon made the decision to use intraoperative ICG-NIR-VA in situations where clinical assessment alone seemed insufficient. This study was approved by the local ethic committee (registration number: 2022–16,484). STROBE guidelines were followed.^17^

### Outcome definition

Data collection included patient demographics, details of free flap reconstruction, relevant comorbid conditions, as well as individual risk factors. Perioperative major complications requiring additional surgical management were systematically documented. Major complications comprised partial flap necrosis, defined as loss of >5% of the distal flap portion remote from the vascular pedicle, complete flap loss with subsequent reconstructive failure, wound dehiscence, hematoma formation, and microvascular complications. Secondary surgical interventions included flap re-advancement, delayed split-thickness skin grafting, or repeat free flap reconstruction. Furthermore, the influence of ICG-NIR-VA on intraoperative flap design and on the extent of excision of poorly or non-perfused tissue was analyzed. Distal or complete flap necrosis constituted the primary study endpoints, whereas the need for reoperation and postoperative length of hospital stay (POLHS) were defined as secondary outcome parameters. Patients were stratified according to the intraoperative method used to assess perfusion, either ICG-NIR-VA (study cohort) or conventional clinical evaluation alone (control cohort). In accordance with safety recommendations, ICG-NIR-VA was not applied in patients with known iodide hypersensitivity, overt hyperthyroidism, thyroid nodules, or autonomous thyroid function.

### Assessment of intraoperative flap perfusion

After flap elevation and isolation on its vascular pedicle, intraoperative perfusion assessment was performed prior to flap division. Mean arterial pressure was maintained at ≥70 mmHg for at least five minutes using cafedrine/theodrenaline (Akrinor®, ratiopharm GmbH, Ulm, Germany). Initial assessment relied on clinical parameters, including capillary refill, skin colour, and bleeding, with the senior surgeon deciding on the need for additional ICG-NIR-VA.

### Indocyanine green near-infrared video angiography

Indocyanine green (Verdye®, Diagnostic Green GmbH, Aschheim-Dornach, Germany) was diluted in sterile water and administered as an intravenous bolus (0.1 mg/kg) via central or peripheral access, remaining within recommended dosing limits. ICG-NIR-VA was performed with the flap in situ and perfused through its native vascular pedicle prior to division and transfer. Perfusion was assessed using the Fluobeam® 800 system (Fluoptics, Grenoble, France), with real-time fluorescence imaging analyzed using Fluosoft™ version 2.3.

### Statistical analysis

Statistical analyses were performed using GraphPad Prism 10.1 (GraphPad Software, Inc., San Diego, CA) and SPSS Version 20.0 (IBM, Armonk, NY). Continuous, categorical, and dichotomous variables were analyzed using the unpaired two-sided *t*-test, Mann–Whitney U test, and Fisher’s exact test, as appropriate. Sensitivity, specificity, and positive and negative predictive values were calculated using 2 × 2 contingency tables. The association between defect size and PSC flap necrosis was assessed using binary logistic regression. All tests were two-sided, with p < 0.05 considered statistically significant.

## Results

### Cohort characteristics

The study cohort comprised 112 individuals who underwent lower extremity reconstruction using a free PSC flap with 45 patients (40.2%) undergoing surgery with ICG-NIR-VA (study group) and 67 patients (59.8%) without ICG-NIR-VA (control group). Both groups did not significantly differ in patient details, comorbidities, or indications for reconstruction. Patient related characteristics are summarized in Table 1. A comparison of operative details between both groups is given in Table 2.

**Table 1:** Patient details, distribution of comorbidities, individual risk factors, indications for reconstruction (ASA = American society of anesthesiologists; BMI = body mass index, n = numbers; SD = standard deviation).

Patient details n (%)	Total	Study Group	Control Group	p-value
Number of flaps (%)	112 (100%)	45 (40.2%)	67 (59.8%)	–
Number of patients (%)	112 (100%)	45 (40.2%)	67 (59.8%)	–
Mean age [years] ± SD	54 ± 19	53 ± 16	56 ± 21	0.39
Mean ASA class ± SD	2.1 *±* 0.5	2.0 *±* 0.5	2.1 *±* 0.5	0.84
Gender [female/male]	57/55	27/18	29/37	0.12

**Comorbidities**				
Arterial hypertension (aHTN)	65 (58.0%)	28 (62.2%)	37 (55.2%)	0.56
Peripheral artery disease (PAD)	33 (29.5%)	16 (35.6%)	17 (25.4%)	0.29
Chronic obstructive pulmonary disease (COPD)	9 (8.0%)	3 (6.7%)	6 (9.0%)	0.73
Chronic kidney disease (CKD)	14 (12.5%)	4 (8.9%)	10 (14.9%)	0.40
Insulin dependent diabetes mellitus (IDDM)	8 (7.1%)	5 (11.1%)	3 (4.5%)	0.26
Mean BMI [kg/m^2^] ± SD	26 ± 5.1	27 ± 4.9	25 ± 5.1	0.88

**Individual risk factors**				
Hypercoagulable state	5 (4.5%)	2 (4.4%)	3 (4.4%)	0.99
Radiation therapy	6 (5.4%)	4 (8.9%)	2 (3.0%)	0.22
Chemotherapy	8 (7.1%)	3 (6.7%)	5 (7.5%)	0.99
Alcohol consumption	11 (9.8%)	3 (6.7%)	8 (11.9%)	0.52
Active smoking	26 (23.2%)	8 (17.8%)	18 (26.9%)	0.36

**Indications for reconstruction**				
Trauma	53 (47.3%)	23 (51.1%)	30 (44.8%)	0.57
Infection	40 (35.7%)	14 (31.1%)	26 (38.8%)	0.43
Tumor	11 (9.8%)	7 (15.6%)	4 (6.0%)	0.11
Burns	1 (0.9%)	0 (0%)	1 (1.5%)	0.99
Scarring	7 (6.3%)	1 (2.2%)	6 (8.9%)	0.24

**Table 2:** Comparison of operative details between both groups (OT = operation time; SD = standard deviation).

Operative details n (%)	Total	Study Group	Control Group	p-value
Number of flaps (%)	112 (100%)	45 (40.2%)	67 (59.8%)	-
Mean OT [min] ± SD	345 ± 91	360 ± 64	337 ± 103	0.23
Mean defect surface [cm^2^] ± SD	185 ± 68	184 ± 80	186 ± 59	0.91
Mean defect length [cm] ± SD	23 ± 4.1	22 ± 4.8	23 ± 3.4	0.39
Mean defect width [cm] ± SD	7.9 ± 2.1	8.1 ± 2.5	7.9 ± 1.7	0.78
Mean flap surface [cm^2^] ± SD	199 ± 71	205 ± 82	194 ± 61	0.44
Mean flap length [cm] ± SD	24 ± 4.4	24 ± 4.1	24 ± 4.7	0.13
Mean flap width [cm] ± SD	8.1 ± 1.9	8.4 ± 2.2	8.0 ± 1.7	0.12

**Recipient Vessels**				
Arteria tibialis anterior	34 (30.4%)	18 (40.0%)	16 (23.9%)	–
Arteria tibialis posterior	62 (55.4%)	19 (42.2%)	43 (64.2%)	–
Arteriovenous loop	13 (11.6%)	6 (13.3%)	7 (10.5%)	–
Arteria fibularis	3 (2.6%)	2 (4.5%)	1 (1.4%)	–

### Comparison of outcomes between the two groups

**Fig. 1:**
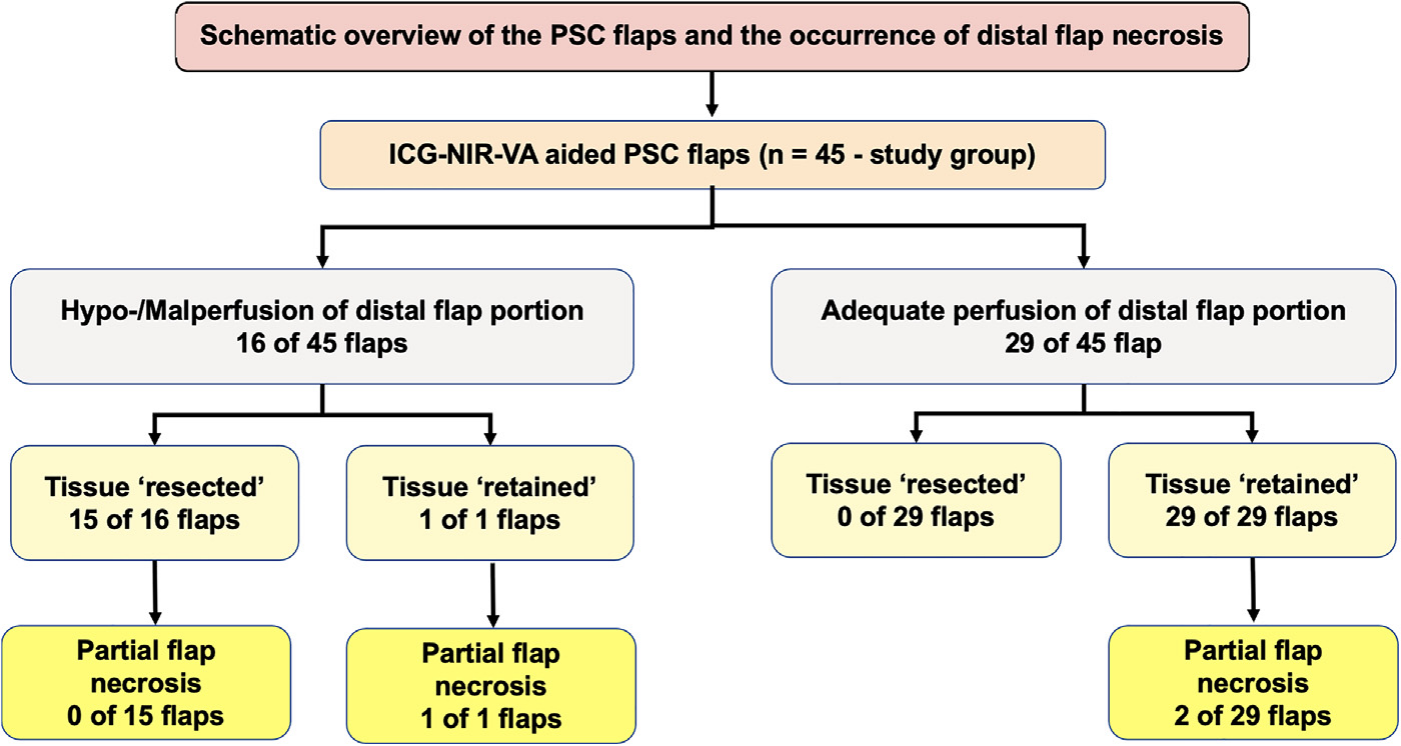
Schematic overview of ICGFA-aided PSC flaps (study group) and the development of postoperative distal flap necrosis.

**Fig. 2:**
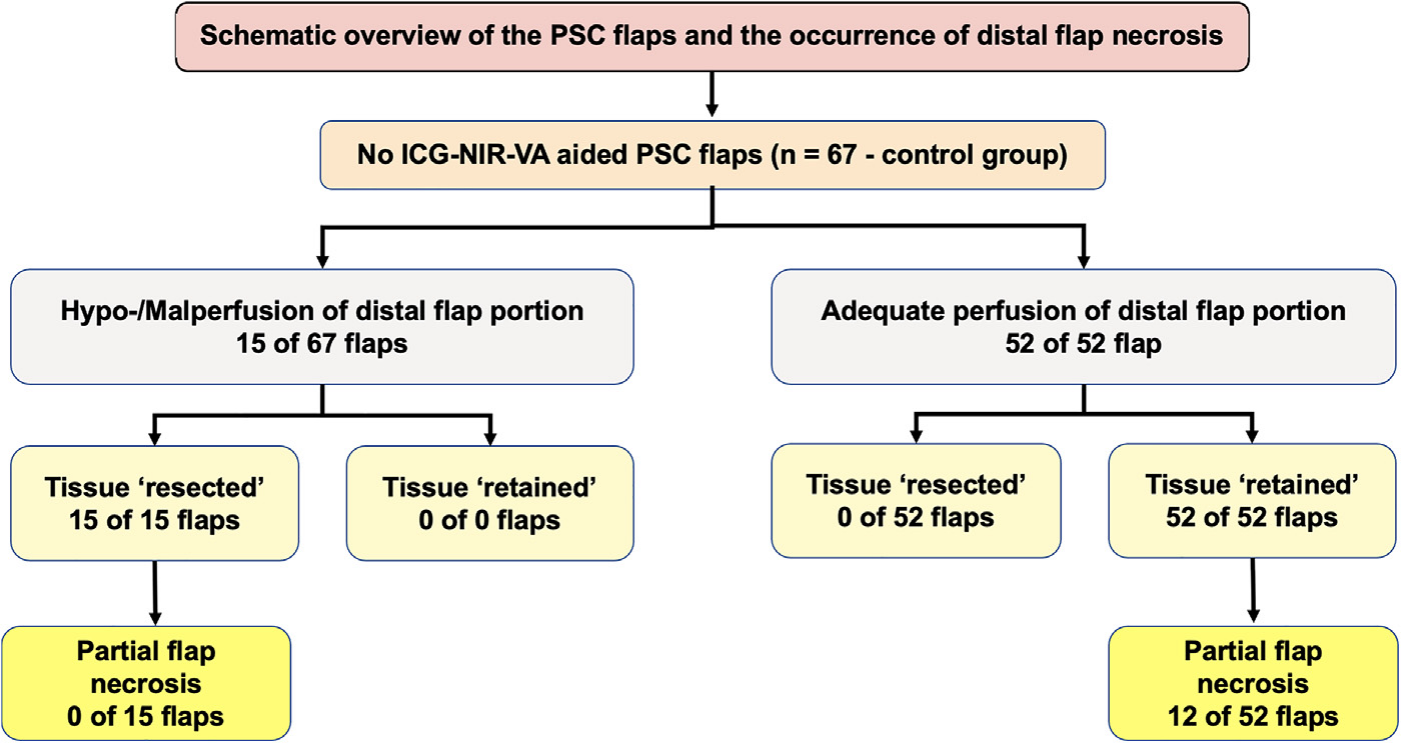
Schematic overview of non-ICGFA-aided PSC flaps (control group) and the development of postoperative distal flap necrosis.

**Fig. 3:**
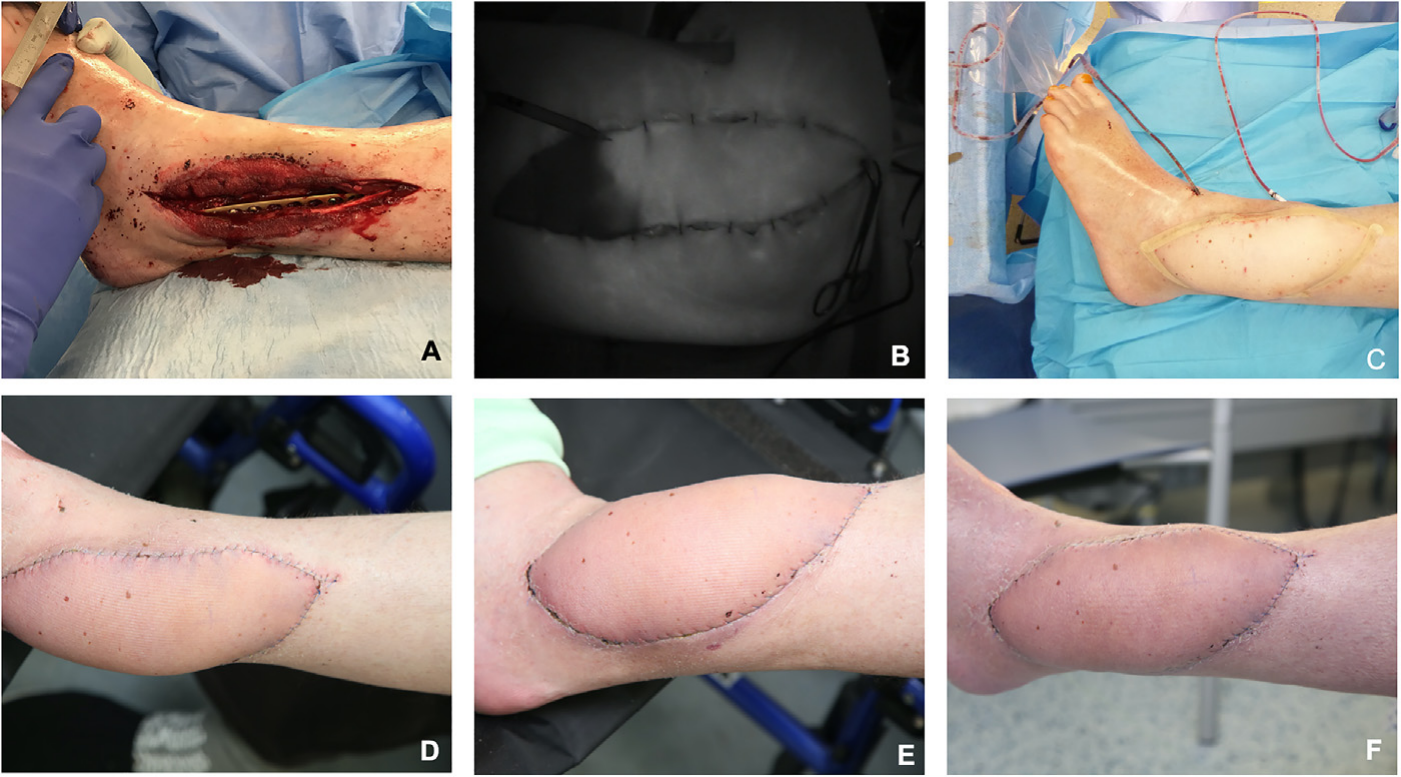
(A) A 60-year-old man presented with a 24 × 7 cm soft-tissue defect over the left medial malleolus with exposed osteosynthesis following a trimalleolar fracture. Reconstruction was performed using a free PSC flap of 29 × 8 cm. (B) Intraoperative ICG-NIR-VA revealed insufficient perfusion in the distal portion of the flap, prompting targeted resection of the compromised tissue. Arterial revascularization was achieved via an end-to-side anastomosis to the left anterior tibial artery, while venous drainage was established using coupler-assisted anastomoses of two concomitant veins (C). The postoperative course was uneventful, and the patient was discharged to a rehabilitation facility 13 days after reconstruction (D-F).

The postoperative course was complicated by partial flap necrosis in three ICG-NIR-VA-aided flaps (6.7%) and in twelve flaps (18.0%) of the control group (OR: 3.1; CI: 0.9 to 10.6; n = 3 (study group) vs n = 12 (control group); p = 0.09). The PSC flaps (6.7%) of the study group received debridement and split-thickness skin grafting (STSG). In comparison six PSC flaps of the control group received STSG (9.0%), five PSC flaps underwent debridement and local flap advancement (7.5%), and one flap with partial flap necrosis led to reconstructive failure and required an additional flap surgery with an anterolateral thigh flap (1.5%). Poor perfusion of the distal PSC flap portion was confirmed by ICG-NIR-VA in 16 cases (35.6%) of the study group. In 15 PSC flaps (33.3%) the corresponding areas were resected, and all flaps healed uneventfully. Thus, ICG-NIR-VA additionally aided decision-making and guided free flap design in 15 out of 45 flaps (33.3%), with no complications occurring postoperatively. Partial flap necrosis occurred in three flaps (6.7%) in the ICG-NIR-VA group. In one case, intraoperative ICG-NIR-VA indicated insufficient perfusion, but the findings were not followed, resulting in partial flap necrosis. In two additional cases, distal flap necrosis occurred despite adequate ICG findings. Additionally, there were four PSC flaps (8.9%) in the study group with intraoperative conclusive inadequate clinical perfusion findings. In all cases, ICG-NIR-VA demonstrated adequate perfusion which prompted us to retain tissue we otherwise would have resected unnecessarily relying solely on clinical findings. These flaps healed uneventfully. Based on these data, the sensitivity of ICG-NIR-VA aided PSC flap perfusion assessment was 94%, and the specificity was 93% with a positive predictive value of 75% and a negative predictive value of 98%. The overall diagnostic accuracy was 93%. In the control group, intraoperative conclusive poor perfusion of the distal flap portion was evident in 15 flaps (22.4%). The corresponding areas were resected in all of these flaps, and all flaps healed uneventfully. 52 PSC flaps showed intraoperative a good perfusion of the distal flap portion (77.6%). However, of these 12 (18%) developed distal flap necroses as described before. Based on these data, the sensitivity of no ICG-NIR-VA aided PSC flap perfusion assessment was 56%, and the specificity was 100% with a positive predictive value of 100% and a false negative predictive value of 91%, and an overall accuracy of 82%. Total flap necrosis was evident in one PSC flap of the study group (2.2%) and two PSC flap (3.0%) of the control group ((OR: 1.4; CI: 0.2 to 20.0; n = 1 (study group) vs n = 2 (control group); p = 0.81). Of these, the patient with total PSC flap necrosis of the study group (2.2%) required amputation, while the other two patients of the control group (2%) received an additional flap surgery with an anterolateral thigh flap (3.0%). The total PSC flap necrosis occurred as a consequence of a fulminant postoperative arterial thrombosis (2.2%) of the PSC flaps pedicle of the study group (n = 1), and a fulminant postoperative combined arterial and venous thrombosis (3.0%) of both PSC flaps pedicle of the control group (n = 2). The salvage procedure has failed in these cases. In addition, another PSC flap of the study group developed arterial thrombosis (2.2%). However, the thrombotic vessel segment was resected and successfully re-anastomosed. Other surgical complications such as wound dehiscence ((OR: 0.6; CI: 0.1 to 2.9; n = 2 (study group) vs n = 5 (control group); p = 0.70)) or hematoma evacuation ((OR: 0.5; CI: 0.0 to 3.4; n = 1 (study group) vs n = 3 (control group); p = 0.53)) did not show a significant difference. ICG-NIR-VA-aided PSC flap reconstructions required fewer additional recipient-site surgeries (9/45 [20.0%] vs. 24/67 [35.8%], p = 0.10). The mean postoperative length of hospital stay was lower in the ICG-NIR-VA group compared to the control group; however, this difference was not statistically significant (19 ± 10 vs. 21 ± 11 days; 95% CI: −2.2 to 5.6; p = 0.40). More comprehensive information is shown in Table 3. A schemantic overview of the PSC flaps and the occurrence of distal flap necrosis is illustrated in Fig. 1, Fig. 2. Exemplarily, a case is shown (Fig. 3A – F), where intraoperative ICG-NIR-VA showed a malperfused distal PSC flap portion. The corresponding area was resected, and the flap healed uneventfully.

**Table 3:** Distribution and comparison of recipient-site surgical complications between both groups.

Comparison of postoperative surgical complications n (%)				
Major complications	Total	Study Group	Control Group	p-value
Partial flap necrosis	15 (13.4%)	3 (6.7%)	12 (18.0%)	0.09
Total flap necrosis	3 (2.7%)	1 (2.2%)	2 (3.0%)	0.81
Wound dehiscence	7 (6.3%)	2 (4.4%)	5 (7.5%)	0.70
Hematoma	4 (3.6%)	1 (2.2%)	3 (4.5%)	0.53
Arterial thrombosis	3 (2.7%)	2 (4.4%)	2 (3.0%)	0.34
Venous thrombosis	2 (1.8%)	0 (0%)	2 (3.0%)	0.24

### Increasing flap length is a significant predictor of partial flap necrosis without ICG-NIR-VA guided PSC flap raising

Mean flap sizes (205 ± 82 cm^2^ vs 194 ± 61 cm^2^; 95% CI: −39.9 to 17.6; p = 0.44) and flap lengths (24 ± 4.1 cm vs 24 ± 4.7 cm; 95% CI: −3.6 to 0.5 p = 0.13) were similar between both groups. Upon comparing flap sizes and lengths of partial distal necrotic and uneventfully healed flaps a significant trend in flap size (261 ± 33.8 cm^2^ vs 186 ± 61.7 cm^2^; 95% CI: −103.5 to – 59.8: p = 0.001) and flap length (29 ± 5.1 cm vs 24 ± 3.8 cm, 95% CI: −9.3 to −3.4; p = 0.003) was seen. Consequently, we ran a bivariate binary logistic regression model to evaluate the association between the use of ICG-NIR-VA and the occurrence of distal flap necrosis. The overall model showed a trend toward statistical significance but did not reach the predefined threshold ((χ^2^(1) = 3.44, n = 112, p = 0.064). Regression analysis demonstrated that the dichotomous grouping variable (“ICG-NIR-VA” vs. “no ICG-NIR-VA”) was associated with increased odds of distal flap necrosis in the no ICG-NIR-VA group; however, this effect did not reach statistical significance (OR: 3.19, 95% CI: 0.84 to 12.01; p = 0.087). In subgroup analyses, increasing flap length proved to be a significant predictor of distal flap necrosis in the control group without ICG-NIR-VA (n = 67; OR: 1.80 per cm increase, 95% CI: 1.29 to 2.52; p = 0.001). In contrast, flap length did not reach statistical significance among ICG-NIR-VA-aided PSC flaps (n = 45; OR: 1.31 per cm increase, 95% CI: 0.98 to 1.77; p = 0.073.

## Discussion

Fasciocutaneous free flaps are routinely evaluated intraoperatively and postoperatively using clinical parameters such as capillary refill after blanching or arterial bleeding at the distal flap margins. However, these assessments are inherently subjective, depend heavily on the surgeon’s experience, and may be influenced by the patient’s hemodynamic status.^18^ Despite reported free flap success rates of up to 99%, even partial flap necrosis can jeopardize the overall reconstructive outcome.^19^ Therefore, accurate intraoperative evaluation of flap viability is essential, yet objective data to guide decision-making in free PSC flap reconstruction remain limited. Recently, we demonstrated that intraoperative ICG-NIR-VA frequently led to modifications in flap design—such as inclusion of additional perforators or resection of poorly perfused tissue—thereby reducing the incidence of postoperative flap necrosis.^20^ Accordingly, we performed this retrospective study to evaluate the influence of ICG-NIR-VA on intraoperative decision-making and PSC flap design in lower extremity reconstruction. Our findings indicate that the principal benefit of ICG-NIR-VA lies in its ability to objectively assess perfusion in the distal segments of the PSC flap, thereby guiding the resection of insufficiently perfused tissue compared with PSC flap procedures performed without ICG-NIR-VA. Notably, the use of ICG-NIR-VA substantially supported intraoperative decisions related to flap design without prolonging operative time and was associated with improved surgical outcomes, including a lower incidence of distal partial flap necrosis and a trend toward reduced major complication rates and shorter postoperative hospital stay.

In our view, dependable intraoperative evaluation of distal free flap perfusion is a key determinant of reconstructive success, particularly in lower extremity reconstruction. This perspective is supported by the systematic review by Li et al. (2018) and the meta-analysis by Smit et al. (2018), both of which identified ICG-NIR-VA as the most effective modality for intraoperative visualization of free flap perfusion and demonstrated its association with improved postoperative flap survival.^15^^,^^21^

In the present cohort, most PSC flaps used for lower extremity reconstruction were performed without ICG-NIR-VA (n = 67), reflecting current clinical practice and corroborating previous studies that emphasize the diagnostic relevance of traditional clinical assessment.^22^ Conventional free flap monitoring remains the cornerstone of postoperative evaluation and is based on inspection and palpation of flap color, temperature, capillary refill, and bleeding characteristics.^23^ Recent analyses by Han et al. and Patel et al. reported accuracy rates ranging from 85 to 95% and up to 93%, respectively, for predicting flap compromise using standard clinical monitoring techniques, thereby confirming their overall reliability.^24^^,^^25^

The ease of use, immediate availability, cost-effectiveness, and non-invasive nature of these clinical parameters make them particularly valuable in time-critical situations and in low- and middle-income settings. Nevertheless, clinical monitoring is inherently subjective and may be affected by lighting conditions, examiner experience, and interindividual variability, especially in patients with darker skin pigmentation, which can limit diagnostic precision.^26^ Moreover, assessment of perfusion in the distal portions of free flaps remains challenging using conventional methods alone. In such situations, objective adjuncts such as ICG-NIR-VA may enhance and corroborate clinical judgment.

The observed discrepancy between clinical assessment and ICG-NIR-VA findings highlights the importance of cautious interpretation of intraoperative perfusion imaging. In addition to timing-related factors and inter-angiosome dynamics, methodological limitations of ICG interpretation must be considered. Various approaches have been proposed to quantify fluorescence signals, including absolute intensity, relative perfusion values, and time-based parameters; however, no standardized method has been established, and each approach has inherent limitations. In particular, relative fluorescence assessment may be influenced by adjacent areas of high signal intensity, potentially leading to underestimation of perfusion in surrounding tissue, as also illustrated in Fig. 3. These factors may contribute to discrepancies between intraoperative ICG findings and postoperative clinical outcomes and support the integration of ICG-NIR-VA with clinical assessment rather than reliance on a single modality.

Our analyses demonstrated reduced rates of distal flap necrosis and major complications, findings that are consistent with previously published evidence supporting the clinical utility of ICG-NIR-VA in the prevention of postoperative flap-related morbidity.^27^ Wang et al., for example, evaluated 22 studies comprising 1021 patients undergoing deep inferior epigastric artery perforator (DIEP) flap breast reconstruction and reported significantly lower rates of fat necrosis and reoperation when ICG-guided flap assessment was applied compared with conventional clinical evaluation (10.9%vs. 21.5%).^28^ Similarly, Bigdeli et al. investigated 88 consecutive adipo- or fasciocutaneous free flaps using intraoperative ICG-NIR-VA and demonstrated excellent diagnostic performance, with a sensitivity of 100% and a specificity of 98.8%, as well as a negative predictive value of 100%. Based on these findings, the authors concluded that ICG-NIR-VA enabled an objective but yet not standardized assessment of flap perfusion and consistently informed intraoperative flap design decisions. In a cohort of 104 patients undergoing head and neck reconstruction with supraclavicular artery island flaps, West et al. reported that ICG-NIR-VA was selectively applied to guide distal flap trimming in 23 cases.^29^ Comparable results were reported by Chen et al., who analyzed 520 free flap procedures in 486 patients, with intraoperative ICG-NIR-VA used in half of the cases. The ICG-assisted group demonstrated lower total flap failure rates (3.9%vs. 8.4%) and a markedly higher flap salvage rate (75.8%vs. 51.4%) compared with procedures performed without ICG-NIR-VA.^30^ In line with these findings, a systematic review by Lohman et al. including 11 studies and 198 free flap reconstructions reported a sensitivity of 90.9% for intraoperative ICG-NIR-VA in assessing flap perfusion, which closely parallels the results of the present study.^31^ Finally, Liu et al. highlighted as early as 2011 that ICG-NIR-VA may further optimize free flap design by facilitating intraoperative perforator localization.^32^ However, we do not consider perforator mapping using ICG-NIR-VA to be the primary indication for its application, as more precise preoperative imaging modalities—such as color-coded duplex ultrasonography and computed tomographic angiography—are readily available and provide superior anatomical detail.^33^^,^^34^ In line with the conclusions of Ludolph et al. (2019), we believe that the greatest clinical value of intraoperative ICG-NIR-VA lies in the assessment of perfusion in the most distal portions of free flaps, where it delivers reliable and reproducible information.^35^ Furthermore, our data demonstrate that the intraoperative use of ICG-NIR-VA was associated with a reduced incidence of partial flap necrosis and, consequently, fewer revision procedures (20.0%vs. 35.8%), although this difference did not reach statistical significance. To our knowledge, this study is the first to specifically evaluate clinical outcomes following ICG-assisted PSC flap surgery with a dedicated focus on perfusion assessment of the distal flap segments. Based on these findings, further prospective studies are warranted to define standardized indications and guidelines for the use of ICG-NIR-VA in PSC flap reconstruction. More recently, several alternative techniques for assessing tissue perfusion have been introduced, including hyperspectral imaging, dynamic infrared thermography, and camera-based photoplethysmography.^21^^,^^36^, ^37^, ^38^, ^39^ These modalities have shown encouraging preliminary results for the non-invasive assessment of cutaneous microcirculation. In this context, Thiem et al. (2021) reported that hyperspectral imaging enabled substantially earlier detection of perfusion deficits—approximately five hours earlier—compared with conventional clinical examination.^36^ In addition, transcutaneous tissue oximetry using an implanted LICOX® probe (Integra Lifescience, Princetown, NJ, USA) has been proposed as a promising method for continuous monitoring of tissue oxygenation, which could be used particularly in the distal portion of free flaps.^40^ Nevertheless, despite their technological potential, these approaches are associated with high acquisition costs, and robust clinical evidence supporting their routine use remains limited.

Several limitations of this study should be acknowledged. First, larger, multicenter studies with randomized group allocation would enhance statistical power and the overall level of evidence. However, prospective randomized study designs may be difficult to implement in this setting, as the use of ICG-NIR-VA is typically guided by intraoperative judgment and perceived uncertainty regarding flap perfusion. While not considered standard of care, surgeons may be reluctant to withhold a potentially useful adjunct in cases of borderline perfusion. The use of ICG-NIR-VA was not randomized but determined intraoperatively at the discretion of the attending surgeon, introducing a potential risk of selection bias. Surgeon-specific factors were not accounted for in the statistical analysis and may represent an unmeasured confounder, although all procedures were performed within a single high-volume center following standardized operative principles. Second, flap harvest was performed by microsurgeons with varying levels of experience, ranging from junior to senior surgeons. This heterogeneity may have influenced operative and flap harvesting times and, consequently, postoperative outcomes. Third, the analysis was limited to lower extremity reconstruction using free PSC flaps, and its generalizability to other anatomical regions or flap types remains uncertain. In addition, it cannot be determined whether tissue deemed hypoperfused by ICG-NIR-VA might have remained viable if left unresected.

## Conclusion

PSC flaps constitute a versatile and reliable option for reconstruction across a wide range of clinical indications. In this study, intraoperative ICG-NIR-VA supported objective assessment of distal flap perfusion and was associated with a lower incidence of partial flap necrosis; however, this difference did not reach statistical significance. In addition, a trend toward reduced reoperation rates and shorter postoperative length of hospital stay was observed, although these differences did not reach statistical significance.

## Ethical approval

This study was approved by the local ethic committee (registration number: 2022–16,484).

## Financial disclosure statement

None of the authors have financial interest in any of the products, devices, or drugs mentioned in this manuscript.

## Funding source

The authors received no funding for data collection or preparation of the manuscript.

## Conflicts of interest

The authors declare no conflicts of interest.
